# Globalization backlands: labor and territory

**DOI:** 10.3389/fsoc.2025.1623871

**Published:** 2025-08-12

**Authors:** Jacob Carlos Lima, Roseli de Fátima Corteletti, Iara Maria de Araújo

**Affiliations:** ^1^Federal University of São Carlos, São Carlos, Brazil; ^2^Federal University of Campina Grande, Campina Grande, Brazil; ^3^Regional University of Cariri, Crato, Brazil

**Keywords:** informality, self-empreneurship, precarius labor, Brazil, labor-territory relationship

## Abstract

This text analyses the relationship between work and territory, focusing on the diverse processes involved in integrating places into the logic of capitalist production, and the role of the state in this integration. Taking the neoliberal policies of the Brazilian state since the 1990s and the inclusion of peripheral territories in the clothing sector’s outsourcing processes as its starting point, it explores the reinterpretation of informal production hubs in a model of individual self-entrepreneurship with cost reduction and increased national competitiveness, on the one hand, and the implementation of policies to reduce labor costs and attract companies to organize outsourcing networks based on the supply of cheap labor and its symbolic value, represented by the offer of formal jobs in a region where these are scarce commodities, on the other hand. Based on two distinct empirical cases, the text demonstrates how informal production hubs are being reinterpreted within a model of individual self-entrepreneurship, which involves reducing costs and increasing national competitiveness. It also shows how policies are being implemented to reduce labor costs and attract companies to organize outsourcing networks based on the supply of cheap labor, as well as the symbolic value of offering formal jobs in a region where these are scarce. The text assumes that the state shapes work territories through regulation, promotion or exclusion, resorting to different policies to this end. In territories traditionally characterized by precariousness, this intervention is perceived as a positive development compared to a previous situation in which employment and income opportunities were limited. This reflects the social, political and cultural relations that shape the space, integrating it into the logic of accumulation. The research was conducted between 2017 and 2019, with the data being updated in 2024. It consisted of exploratory visits to cities and production workshops, as well as interviews with owners and workers of these places.

## 1 Introduction

This article aims to analyze the labor-territory relationship in the process of capitalist modernization in the global periphery and how countries and regions are incorporated into market flows based on: flexible forms of production implemented through public policies to attract investment; mobilization of cheap labor in peripheral regions; resignifying precarious forms of labor and modifying the regulation of capital-labor relations. The aim is to demonstrate the construction of productive networks aimed at reducing labor costs, through different forms of outsourcing. The research question is as follows: to what extent do these processes redefine peripheral territories by integrating them into the market economy in a historical context of neoliberal economic transformation? This can be seen in state policies implemented to attract investment, where labor costs play a central role, as well as in the promotion and support of spontaneous forms of production and labor organization. These were originally geared towards producing and consuming popular goods, but are currently valued for their functionality given the flexibility with which they are organized.

The clothing industry is an example of this process which, linked to the fashion industry, makes it difficult to standardize production processes and, given the ease of opening and organizing production units, enables their territorial diffusion using formal and informal work, home work, and the combination of urban and rural work. This means its global spread in peripheral countries and even in the center of large cities in the so-called developed world (Portes et al., 1989).

This article analyzes two emblematic models of this situation in the Brazilian northeast, a region considered the poorest in the country and subject, since the 1950s, to developmental policies, without great success. The first model examined is a clothing production hub in the state of Pernambuco, the result of work and income strategies found by the local population, mainly women, through the production of popular consumer clothing, sold at popular fairs in the region. Marked by informality, it was ignored by state policies for a long time. From the 1990s onwards, the dynamism of this informal hub was considered an example of the strength of entrepreneurship among the poor, and began to receive official policies to support the entrepreneurial spirit of workers-entrepreneurs.

The second model we are going to address is a government program to support the organization of small production units in the state of Rio Grande do Norte, close to Pernambuco, to act as outsourced companies for large national and international companies and store chains. In these units, regular salaried work predominates, in small towns and even districts of the region known as Seridó.

They also represent different forms of State intervention in the implementation of liberalizing/flexible policies, originating in the 1990s with the expansion of production outsourcing incorporating new peripheral territories that were outside the regular circuit of goods production. We can say that the expansion into new territories takes advantage of the pre-existing productive context, previously perceived as backward and now seen as exemplary of neoliberal modernity. The programme was implemented in 2013.

This decade represented the country’s insertion into the new dictates of globalized capitalism: opening of markets to the import of goods, productive restructuring, denationalization of industrial sectors, strong economic recession, and rising unemployment. This situation only changed during a brief period of time, from 2003 to 2014, marked by significant economic growth, an increase in formal employment, and a reduction in social inequalities, in the wake of the rise of popular governments and the expansion of public policies. This situation continued until the economic crisis of 2014, which was deepened by the parliamentary coup of 2016.

In any case, whether with right-wing or left-wing governments, the neoliberal macroeconomic logic was maintained throughout this period with the increasing flexibility of labor relations and the respatialization of productive activities in search of lower costs. This process begins to favor global production chains in outsourcing networks, in which the cost-labor relationship becomes an important variable in the displacement of production sectors across regions, countries, and continents. New information and transport technologies reduce the weight of location in the final cost of production and the State uses tax wars between subnational territories in search of investments, in a context of formation of a global market, in which borders lose importance.

In the context of labor, outsourcing networks and the search for cheap labor pressure the elimination of social obligations that increase labor costs and are considered obstacles to global competitiveness. Loss of social rights, career opportunities, and job stability are the problems that accompany the “modernization” of labor relations and “reforms” in several countries. The neoliberal discourse encourages the individualization of labor relations and the worker becomes responsible for his/her employability, having to be active, entrepreneurial, flexible, and mobile. This process, however, is complex and the territory plays a fundamental role in the new configurations or resignifications of work.

According to Harvey (1992), work is embedded in the logic of capital, which requires valuation and circulation. This constitutes a social practice that is mediated by institutions, cultural forms and economic structures that are situated in time and space. Territory is not only physical support, but also a field of struggle (Bourdieu, 2013), in which work is organized, fragmented, reinvented, and re-signified based on social relations. For Santos (1999), territory reflects power relations that determine the use and control of space, articulating techniques, norms, and policies. In other words, it is an instance of the reproduction of production relations (Lefebvre, 1991). The state shapes territories of work through regulation, promotion or exclusion. In the Brazilian case, this makes informality structural, whether in metropolises and large cities or rural areas (Maricato, 2017).

The territorial mobility of capital refers to its ability to move geographically in search of better appreciation conditions, exploiting spatial asymmetries in terms of costs, regulations, labor, and resources (Wallerstein, 2012). Indicators of this mobility include production costs (wages, taxes and inputs), state incentives, transport infrastructure, logistics, technology and proximity to consumer or supplier markets. In the cases presented here, given the labor-intensive nature of the production sector, labor costs and regulations, as well as state incentives, dominate, in contrast to technological issues and proximity to large centers. Given the country’s size, transport logistics ultimately compensate for distance from consumer and supplier markets. In other words, this mobility and its advantages must be contextualized.

Both models represent experiences in reducing labor costs, whether in terms of income through salaries paid or in terms of social rights, which are non-existent or have reduced costs, in a region marked by an abundance of labor and a restricted job market. Both situations reflect previous historicities, now resignified. They are places marked by declining agricultural activities that assume new prominence, with labor-intensive industrial activities, which, at first glance, have become alternatives to poverty and prevailing unemployment. However, upon closer analysis, it is possible to verify an environment not of precarious work, but of the precariousness that constitutes activities carried out informally, or of public policies that benefit business networks, to the detriment of labor, however, it should not be eliminated.

The text is organized around the following topics, in addition to this introduction: The research details its operationalization and the techniques used. Item 2 discusses the Sulanca fairs and the organization of an informal clothing manufacturing hub. This item has two sub-items: ‘From artisanal production to the clothing manufacturing hub’ and ‘Before and after the pandemic’. Item 3 presents the second case study in the Seridó region of Rio Grande do Norte. This item includes a sub-section on the circuits and production flows in sewing workshops. The text concludes with considerations on the two cases and analyses of what unites and separates them. It also considers the different forms of the same process: the reconfiguration of peripheral territories and their inclusion in the globalized economy.

## 2 The field research

The article is based on empirical research conducted in the region between 2017 and 2021, with an update in 2024. The research involved collecting secondary data on the regions and municipalities studied, visiting production units, conducting non-participant observations and interviews with small landowners, entrepreneurs and workers, and reviewing literature on the subject. Seven visits were made to the municipality of Santa Cruz, where a total of 15 interviews were conducted. Of these, nine were with women seamstresses who own informal sewing workshops; three were with small, formalized entrepreneurs; one was with a jeans laundry manager in the neighboring municipality of Toritama; and one was with the Director of Economic Affairs at the Secretariat for Economic Development of the City of Santa Cruz. Working conditions in sewing workshops and at points of sale in the Fashion Shopping Center and on the promenade were also observed. During the pandemic, a further 12 interviews were conducted with home-based seamstresses via WhatsApp.

All interviews were arranged in advance via contacts with students from the Federal University of Campina Grande who reside in the area, as well as with researchers who have conducted or are conducting research at the Polo. The interviews were recorded and later transcribed. Photographs were taken of the environment, the machinery used and the items produced. The interviewees were informed of the academic nature of the research, and fictitious names were used to protect their identities and comply with ethical research standards.

Unlike the Santa Cruz clothing hub, which has been the subject of several studies, the “Mais Sertão” Programme of the Rio Grande do Norte state government has received little analysis and is therefore not well known, given its more recent establishment. Consequently, the research was more exploratory in nature and encountered greater challenges in obtaining primary data.

The research involved collecting secondary data on the Seridó region, the area with the highest concentration of sewing workshops among its 25 municipalities. This was supplemented by visits to production units, non-participant observation and interviews with workshop owners and community leaders. A unit in a rural area of one of the municipalities was also included.

We made four visits to the Seridó Potiguar region, basing ourselves in the city of Caicó for initial contacts and mediations. Caicó is one of the region’s main cities and is home to a branch of the Federal Institute of Rio Grande do Norte. This was established in 2009 as part of the government’s Plan for the Expansion of the Federal Network of Professional, Scientific and Technological Education. The institute offers a technical course in clothing manufacturing and was established in Caicó with the aim of training workers for clothing production units, which increased in number in 2013 with the Pro-Sertão Programme. At this institution, we received mediation and information about the operation and location of sewing workshops. As IFRN offers a technical course in clothing, it has gathered information about this sector. We also visited the cities of São José do Seridó and Jardim do Seridó.

In São José do Seridó, we interviewed the owners of a company which is one of the region’s main outsourcers. We also visited a rural area of the municipality, where we interviewed local leaders and a faction operating in the community. In Jardim do Seridó, we interviewed two faction owners before going to the rural area to conduct another interview with the owner of the community faction. We also interviewed owners of factions in sewing workshops in both the urban and rural areas of the two municipalities. From the initial contacts and the network of relationships established, referrals occurred naturally. After the pandemic, we confirmed that the factions we had visited were still operating, albeit without any return.

The selection of the two cases was not intended to be comparative, but rather to illustrate the formation of clothing production hubs in small regional interior towns, which operate within a flexible accumulation regime, as described by Harvey (1992). Based on this characterization, we demonstrate how state action has given new meaning to these spaces within this accumulation logic.

We analysed the data considering the specificities of the cases and the interviewees’ discourse in relation to the theoretical framework used.

## 3 Sulanca fairs—self-entrepreneurship

We began by analyzing a spontaneously formed clothing production cluster, outside of any state umbrella, located in the region known as Agreste Pernambucano, marked by informal production, ignored for a long time by official statistics, and seen as a clothing production aimed at the low-income population. Initially, the clothes were sold by the seamstresses themselves at weekly fairs and purchased by local street vendors. Later, local producers began to travel to other states in search of new markets, which boosted production growth. From the 1990s onwards, this cluster was classified as a “clothing hub,” a successful case, representing the entrepreneurship of workers in the region, outside of formal wage relations and with the potential to compete against imported articles, even with low technological investment. Currently, it represents the second largest clothing production hub in the country, surpassed only by the metropolitan region of São Paulo.

This decade, in Brazil, was marked by neoliberal economic policies with the opening of the national market to the import of products and the reduction of state protection for national industry, which caused substantial changes in the Brazilian industrial structure. Sectors were closed or denationalized, public companies were privatized, leading to an exponential increase in unemployment and informalization in labor relations. This informalization, with a significant presence in our labor market, reached levels close to 70% of the entire employed workforce. Initiatives to make labor relations more flexible were also implemented through projects to deregulate labor and reduce social protection for workers, which were seen as things that increase costs and prevent Brazilian products from becoming more competitive abroad. Although restricted in their final results, changes in legislation allowed the outsourcing of so-called core activities, and measures such as temporary work, time banks, among others, led to a weakening of collective and union organizations.

In their quest to understand how capitalism renews itself, Boltanski and Chiapello (2020) identify characteristics that can be analysed as the ‘new spirit of capitalism’. Updating Weber’s concept of the Protestant ethic and the spirit of capitalism from the perspectives of culture and morality, and taking into account the structural aspects identified by Marx, they demonstrate how ideologies formed by beliefs associated with economic activities evolve in accordance with each historical period. From this perspective, Lima (2010) notes a change in the perception of work and the values associated with it, as well as its collective nature and potential to form identities and social projects. He states that it is important to understand how values are constructed and transformed through social and work relationships. A growing example of this is the reinterpretation of informality as entrepreneurship and flexible work. This ideology has gained increasing prominence alongside neoliberal advances, which aim to reduce the state’s role in mediating capital-labor relations and ‘privatise’ social policies by outsourcing social services to companies and social organizations.

Informality, now reinterpreted as flexible work, has left its mark on the job market in a country that only abolished slave labor in 1888. The new present in the resignification is in what was previously seen as synonymous with backwardness and social marginalization (Nun, 1989). This is now seen no longer as something that belongs to a poor country, but as something that is potentially formed by entrepreneurs. Paraphrasing De Soto (1987), the problem in Latin America would not be the lack of a State, but its excessive regulation, which would inhibit the entrepreneurial character of the population.

This discourse becomes dominant from then on, and entrepreneurship begins to justify all attempts to “modernize” labor relations, in other words, their deregulation. From 2003 onwards, with the arrival of popular governments to power, there was a significant improvement in the formalization of the labor market with policies that, even without substantially changing the neoliberal logic, mitigated its consequences with the implementation of public policies aimed at generating formal jobs and the search for the formalization of informal activities, such as the MEI (Individual Micro Entrepreneur) program, created in 2008 and developed from 2009 onwards (OIT, 2014). The figure of the entrepreneur becomes increasingly central in public employment policies.

The implementation of this program had repercussions from the point of view of the formalization of labor relations in the region of Agreste Pernambucano production hub, since many owners of small production units and workshops saw in the program a possibility of leaving informality and becoming formalized entrepreneurs, with low costs. However, around 70% of activities remain informal. Even before, in the 1990s, the discourse of entrepreneurship began to be disseminated by SEBRAE, which saw the hub as a real possibility of competitiveness against Chinese products that were invading the clothing market, given the low costs (Lima, 2002).^1^

### 3.1 From artisanal production to the clothing hub

Santa Cruz do Capibaribe, Toritama, and Caruaru are the main municipalities that make up the Clothing Hub of Agreste Pernambucano, which began with the production of patchwork quilts and women’s underwear in the second half of the 1950s (Milanês, 2015; Lima, 2002). This hub was established as a survival strategy developed by the local population, mainly women, to face the crisis caused by droughts in a region whose economy, at the time, depended basically on the cultivation of cotton and subsistence agricultural products.

The pioneers in the creation of clothing-related activities began to produce them using scraps of “helanca” as raw materials, a synthetic fabric 100% made from polyamide. These scraps were brought from the southern region of Brazil by truck drivers who were carrying agricultural products produced in the northeast. Regional production began to be sold at the “Sulanca fairs,” which became known for selling cheap clothing aimed at the population with low purchasing power. Held since the end of the 19th century, the traditional Caruaru fair, a large regional market for the exchange of goods in general, began selling locally produced clothing in the 1950s. The expansion of sulanca production to neighboring municipalities such as Santa Cruz do Capibaribe and later Toritama, in the early 1970s, formed an axis of “early morning” fairs through which the street vendors followed in search of low-cost clothing (Lima, 2002; Véras Oliveira, 2013).^2^

The success of the fairs attracted investors from several states in the country. Many people from neighboring cities began to move there in search of work and income, settling mainly in the municipalities of the Hub. This process led to an intensification of clothing production, driving the opening of small informal production units, generally called *facções*^3^ and *fabricos*.^4^ In these spaces, production is most often structured in living rooms, garages, balconies or other rooms in the home of the person who sews.

Subsequently, local manufacturers began to travel to other states and regions in search of new markets, which boosted economic growth in the region. Furthermore, with the growing expansion of productive and commercial activities, the “Sulanca fairs” and “Early morning fairs” needed to be modernized. To this end, large commercial centers or popular malls were built in these municipalities as a way of organizing the thousands of stalls that were spread across the city streets.

Among these, the “Moda Center” in Santa Cruz do Cabiparibe stands out, which has more than 10 thousand commercial points organized into six sectors, which are represented by six colors, as well as food courts, hotels, dormitories, pharmacy, outpatient clinic, and parking for more than six thousand vehicles. The fair is always held on Mondays and Tuesdays, from 7 am to 6 pm, but it is busier on Mondays. Currently, the place receives up to 150 thousand customers per week, and the clothes displayed in the windows for sale follow a general fashion pattern, with styles, colors, and types of fabrics that usually appear in television soap operas. However, at the back of the space where the Moda Center operates, there is still a Sulanca Fair, in the old format and in more precarious conditions. Vendors who were unable to purchase a sales booth inside Moda Center resort to this external space, the “*poeirão*” or “calçadão,”^5^ as it came to be called after renovations in 2014, to sell their clothing at even lower costs, reaching popular consumers who can only afford this type of product. However, there are cases of vendors who own boxes inside Moda Center, but maintain stalls on the street, or in the most precarious area of the commercial center.

The production model used in the Hub, in addition to encompassing the three main cities in the Pernambuco Agreste region, has also been expanding and moving production units to rural areas. This factor has become increasingly common and occurs both due to the difficulty of finding qualified seamstresses in urban areas (since most of them already have jobs), and also aims to reduce production costs, as contractors generally pay rural residents lower prices for sewing pieces, compared to city workers.

It is worth noting that, even with the economic dynamism generated by this hub and its insertion in global dynamics, the maintenance and reproduction of traditional characteristics may be observed both in the way of producing and marketing clothing. Informality and precariousness in labor relations and conditions appear in the way production is carried out—home-based production units that mobilize a family workforce, cramped environments, generally adapted houses.^6^

Opening a *fabrico* or *facção* by their own is a dream for many residents of this region, since becoming an entrepreneur is perceived and encouraged by official propaganda as something dignified and innovative, ingredients that help to raise the status and self-esteem of the owners. Informality is seen as advantageous for workers, as it allows them to earn greater profitability, since the business owner does not need to pay taxes or social rights to workers. Most people who work with sewing have the desire to open their own business, produce their own pieces, and create a brand, aiming to obtain more freedom and autonomy in the work sphere (Corteletti and Milanês, 2021). Many producers also sell their goods, having boxes at the Moda Center, where the products are displayed. This display guarantees that orders, once settled, are dispatched to the destination determined by the buyer.

According to Milanês (2015), it is possible to see that this desire to “work without a boss” has deep roots in the family context of the individuals who make up the Hub, as well as in the historical experience as rural workers, small business owners, and ranchers. This configuration, in turn, although it represents autonomy for some, results in the imprisonment of others, such as workers, who work exhausting hours and do not have any labor rights or social protection. Thus, it can be seen that the much-dreamed-of autonomy and freedom in the work environment is quite relative, considering that, in the case of seamstresses, the productive work carried out at home is mixed with reproductive work, leading them to work up to 16 h a day.

Although the productive and commercial activities of the Hub should be considered as opportunities for income generation and social reproduction strategies, this market dynamic also has its price. On one side, there are employers seeking flexibility, cost reduction, and risk transfer in a competitive environment, and, on the other, there are seamstresses and shopkeepers, women, mothers, and wives, who combine the dual role of taking care of the home and contributing to their livelihood in the same physical space, without protection of labor rights, without access to maternity leave, unemployment insurance, retirement, vacations, among others.

Abreu and Sorj (1993), analyzing the home-based work of seamstresses, state that, although there are differences in its configuration around the world, one of the most striking characteristics of this form of production is that it is an essentially female activity. It occurs because home-based work “has always been based on domestic work and the sexual division of labor, both in the sphere of production and reproduction (p. 23, our translation).” Furthermore, home-based work ends up being more advantageous for women, as it offers the possibility of conciliating an activity that generates income with domestic activities, such as caring for children, preparing food, and organizing and cleaning the house.

Taking these elements into account, Neves and Pedrosa (2007) observe that all configurations of precariousness caused by informality are in line with the logic of the textile sector, since the forms of restructuring in the clothing industry, instead of incorporating new technologies, end up being guided much more by the decentralization and flexibility of production, with the main objective of increasing productivity and reducing costs. In this sense, outsourcing networks, subcontracting, and home-based work, in addition to being inherent to the dynamics of clothing manufacturing, are revitalized today, in the face of a process that maintains continuous relations between the formal and the informal, moving productive activities to other spaces and transferring to the workers some expenses necessary for the manufacture of goods.

With the proposals to formalize production units through the MEI Program, new market possibilities emerged, expanding the sale of clothing. Furthermore, workers informally employed in *fabricos* and *facções* also have seen the Program as an opportunity to become formal entrepreneurs, with their own business, since there are no other employment possibilities in the region. However, some limitations were imposed. As the program allows the hiring of only one employee, recent research indicates the continuation of a complementary relationship between formal and informal work, since it is unlikely that the entrepreneur will maintain his/her *facção* with only one employee hired, which ends up resulting in new informal work contracts (Pereira, 2018).

Although the city is perceived as an entrepreneurial hub, the nature of work in the clothing industry highlights contradictions within the entrepreneurial discourse. Firstly, there is an emphasis on autonomy, which opening a workshop enables in theory. However, there are caveats because, in many cases, this autonomy is accompanied by explicit subordination to existing forms of outsourcing and dependence on orders. Secondly, there is the issue of the intense work, long hours and earnings that do not always exceed the formal minimum wage. Consequently, not all interviewees consider themselves entrepreneurs, despite the fact that this discourse, amplified by state and private bodies, has been internalized in terms of individualization and the idea that success results from individual effort and the absence of bosses.

### 3.2 Impact of the pandemic on the work of seamstresses in the hub

The arrival of the new Coronavirus in Brazil, in the second half of March 2020, brought with it an unprecedented health collapse, which intensified a social, political, economic, and humanitarian crisis, which mainly affected the most vulnerable social groups in the country, such as low-income people, informal workers, the black population, indigenous people, and women.

During the pandemic, it was observed that the productive work of clothing manufacturing, instead of decreasing, was intensified. The demand for fabrics increased so much that the input was in short supply in stores in the city of Santa Cruz do Capibaribe, which caused the prices of this raw material to rise, causing an increase in the production costs of clothing. It was found that some seamstresses were “stopped” during the first months of the pandemic, others began producing masks as outsourced workers for medium-sized businesses and/or for larger companies, which had a contract with the Government of the State of Pernambuco. Finally, some seamstresses took advantage of the time they were “stopped” and of the money from emergency aid^7^ and started producing their own clothing items, to be sold at the fair or online (Instagram or WhatsApp).

Although sewing and housework are closely linked, it is difficult to calculate how much time they dedicated to each activity. Many seamstresses stated that what changed most in their work routines during the pandemic was the increase in working hours, which could last from 12 to 16 h a day, in order to meet the demands, a situation that was also common in pre-pandemic times. They stated that they were no longer able to establish a fixed schedule for productive activity, as they worked at night and on weekends. Furthermore, childcare has also intensified, as previously children spent part of the day at school or in daycare, with the quarantine everyone had to stay at home and many had difficulty accompanying their children in remote school activities.

Finally, we realized that the pandemic had exacerbated the lack of limits on working hours, meaning that seamstresses could no longer establish fixed working hours, as they were working day and night, including weekends. One interviewee reported that she could no longer take Sundays off because she now works intermittently.

It is worth noting that, in the cities of the Pernambuco hub, there is a lack of seamstresses to work in clothing factories and unemployment is practically non-existent in the region, with a strong cultural characteristic focused on promoting self-employment and self-entrepreneurship predominating, which was reinforced by the far-right government that ruled the country between 2019 and 2022.

## 4 The Seridó region of Rio Grande do Norte and outsourcing networks

Our second case is located in the region known as Seridó Norte Rio-grandense, marked by experiences of industrial outsourcing of national clothing factories and international brands with government mediation. However, sewing workshops, in the current context, do not constitute the first outsourcing experience in the countryside of Rio Grande do Norte. According to one interviewee, a businessman from the municipality of São José do Seridó, who arrived from Rio de Janeiro, set up a clothing company in the 1990s. He was the one who brought the first company to outsource its production in Seridó and, from then on, the practice began to spread to the region. In 2001, a factory was founded by a local group to produce knitted shirts and later jeans, which gave rise to several workshops.

Another example is the hatmaking sector, concentrated mainly in the cities of Caicó and Serra Negra do Norte, which, since the 2000s, has been outsourcing to well-known brands. The region became the largest producer in the country. In the last decade, due to Asian competition, it has significantly reduced its production, although it is still a reference in the sector (Lins, 2011).

The Seridó region is a territorial district formed by twenty-five municipalities, located in the south-central part of the state of Rio Grande do Norte. Caicó is the most prominent city and main commercial center, 221 kilometers away from Natal, the state capital. In the middle of the semi-arid region, its economy was anchored in agriculture, cotton farming, mining, and subsistence farming. Since the 1980s, it went through a crisis that began in cotton farming, hit by the boll weevil plague, followed by the mining crisis with the fall in the price of ore, and the cooling of the agricultural sector due to constant droughts, which has led the region to decline (Morais, 2005).

This uninspiring context and the search for alternatives for work and income generated local initiatives linked to cultural and identity devices, linked to the knowledge and practices of the region, which allowed a redefinition of its socioeconomic structure, provoking a process of reinvention (Araújo, 2000).

Among the strategies used, the textile segment and the production of goods such as caps and household linen stood out in the 1990s, intensifying the emergence of small manufacturing units and sewing *facções*. A “tradition” is created in the region, in government discourse, of a textile “vocation” that has been configured, in recent decades, in the organization of small sewing workshops that operate outsourced to large companies. In the 2000s, the number of *facções* grew considerably, making Seridó the main investment hub in the clothing sector in Rio Grande do Norte, concentrating the largest number of *facções* in the state and becoming one of the main sources of employment and income for the local population.

As a result of the socio-spatial and productive restructuring of the textile and clothing sector that seeks greenfields in the country, Seridó has been an emblematic example of industrial relocation experiences and outsourcing networks with the support of state policies, through tax incentives aimed at reducing production costs in order to compete with imported products.

The existence of an abundant workforce constantly struggling against unemployment and poverty, as well as the droughts that plague the region, making regular agricultural activities unviable, is what makes it vulnerable to experiments seeking cheap labor. Initially, the focus on sewing workshops is on job creation, which, although unstable, depending on the seasonality of orders, guarantees employment for a population that has no other alternative way of making a living.

Large business groups have found little difficulty to outsource production in the semi-arid region, due to the availability of labor and low costs. However, the obstacles encountered in making it viable, such as training and qualification, in addition to infrastructural problems for its implementation, have been overcome by government initiatives mobilized to meet business demands. Two of them deserve to be highlighted: the creation of IFRN (Federal Institute of Rio Grande do Norte) and the state government’s Pró-Sertão Program.

The IFRN was installed in Caicó in 2009 with a training line for professionals in the textile and clothing sector, with the intention of qualifying workers for the region. Meeting local demands, it offers a technical course in clothing, the only one in the state and the second in the Northeast region, a higher education course in textile production, as well as training in electrical engineering for machine maintenance.

Pró-Sertão is a program of the State of Rio Grande do Norte aiming at developing the textile industry in the countryside, guaranteeing tax incentives and support to develop the textile production chain. Implemented in 2013 by SEDEC (Secretariat of Economic Development), it works in collaboration with FIERN (Federation of Industries of the State of Rio Grande do Norte), SEBRAE (Brazilian Macro and Small Business Support Service), SENAI (National Industrial Training Service), BNB (Bank of Northeast Brazil), and the state textile chain. The program was created with the intention of decentralizing textile production from the metropolitan region of the capital, Natal, to inland cities. In Seridó, it found favorable conditions due to the region’s “textile vocation.” Among the intentions of Pró-Sertão, one is to transform Rio Grande do Norte into a national clothing hub, in addition to creating jobs and installing new workshops and creating its own brands, linked to the priority of serving large textile and clothing factories with headquarters in the state (or even outside it) that have an impact on the economy, with emphasis on the institutional arrangements implemented by cooperation policies between public and private agents (SEDEC-RN, 2015).

The Program emerged in a context of increased demand from large retail chains, three of which are the main suppliers to the *facções* in the semi-arid region of Rio Grande do Norte. A local group with national operations is the main supplier to the *fccções* and the main beneficiary of the creation of Pró-Sertão (SEDEC-RN, 2015).

### 4.1 Production circuits and flows in sewing *facções*

The impacts of the emergence of sewing *facções* in the backlands of Rio Grande do Norte have been highlighted ever since. Owners and workers prefer to use the term “sewing workshops,” as they believe that the term *facção* carries a very negative symbolism due to the criminal factions widespread in the country.

Both workers and owners see the Pró-Sertão program and the proliferation of sewing workshops as gifts because, in a context of job shortages, they are not able to find any other viable alternative. In terms of labor relations, the permanent supervision of the MPT-RN (Public Ministry of Labor) has maintained the consistency of regular contracts. All workers are formal, registered, receiving the minimum wage, but they face instability in orders from partner companies.

In the research, we focused on São José do Seridó and Jardim do Seridó, small cities with populations of 4,558 and 11,655 (IBGE, 2022) respectively, which have the highest concentration of sewing *facções* in the state. They are responsible for the population’s main source of income, making them an emblematic example of modernity represented not only by the industrial work represented by sewing workshops, but also by the dominance of formal work, something rare in the region.

In São José do Seridó, there are 18 sewing workshops, employing around 700 people. 17 of them are located in the city and one is in the rural area, in the Caatinga Grande community. Seven of these workshops belong to the same local group. The owner tells us that the city no longer has enough labor and, for this reason, other workshops of the group were set up in other municipalities in the region.

According to the owners interviewed, their biggest dream is to create their own brands to be less dependent on outsourcing activities. However, they report great difficulties involving the need to aggregate other areas of knowledge, such as developing a collection, modeling, cutting, purchasing inputs, and selling production. A local group from São José do Seridó, in partnership with the IFRN in Caicó, has a textile laboratory with a cutting sector and has managed to launch several of its own brands. This group, however, is different from the others because it has a more consolidated business structure, its own brand, and preferential relationships with the large company that outsources its production in the region. This initiative prevents idleness in the months when companies reduce or even stop clothes distribution for marketing reasons. Another used strategy is to attract seasonal clients by serving companies in other regions of the country.

The workshop that operates in the rural community of Caatinga Grande is also part of the group. There are 63 families in the area who live off agriculture and embroidery. The idea was to absorb the second generation, which was idle due to lack of incentives in the field, droughts, and the closure of the embroidery cooperative. Around 34 young people worked in the workshop that operated in the building that previously housed the local embroiderers’ association.

In Jardim do Seridó, 23 sewing *facções* have already been set up by several local entrepreneurs, employing around 800 people. Two of them are located in the rural community of Currais Novos.

One of the owners of one of the *facções* established in the Currais Novos community used to be an embroiderer. With the arrival of the *facções* in the city, she and her partner began to work in sewing. Through the *facção* owner, in 2017, they benefited from a federal project linked to SENAI, CNPq (National Council for Scientific and Technological Development), and IMTECOT (Implementation of Technologies in Cotton Farming in the Semi-Arid Region), executed by SENAI and under the coordination of CETCM (Centro de Educação e Tecnologias Clovis Motta). They received machines and training at SEBRAE and SENAI.

After 5 years, the project was not continued, so they set up the first *facção* in the community, which came to operate in the old local embroiderers’ association. They soon built their own building and set up the second *facção* in the city, which began to be administered by her partner. The 34 employees who work at the community unit are relatives and acquaintances, mostly women. There is a great tradition of embroiderers in the community and the lack of incentives and difficulty with sales motivated many women to migrate to sewing.

They started working with jeans, but were about to start working with viscose pieces. The owner says that viscose is lighter, whereas jeans is too heavy and tiring for women working all day long. The interviewee’s partner set up his workshop in the city with 45 workers, 23 women and 22 men. He claims that there is no distinction between genders in the work. Most have high school degrees and many are already in higher education. He is thinking about creating his own brand and is in the planning phase. He believes it is a way to prevent the lack of supply from companies that outsource production.

Sewing has always been considered a feminine skill, a desirable activity for women, included within their home and family care skills. When referring to the sexual division of labor, Hirata and Kergoat (2003) warn of the need to not simply stop at the observation of inequalities, but to perceive their systematic nature assuming conjunctural and historical forms. Differences are hierarchized, creating a gender system. Asymmetries are perceived in salaries, work discipline, and criteria that define the qualification of tasks. In Seridó, sewing is carried out by both sexes, although women predominate, and this is where precariousness is most common.

In a study carried out in clothing production cooperatives in Northeastern Brazil, Lima (2002) highlighted the strategies of companies in recruiting labor involving men and women, sometimes reinforcing stereotypes and reaffirmed beliefs around the sexual division of labor, sometimes breaking them according to the convenience of production and profit. At certain times, women were considered ideal because they were considered flexible, as they only worked when companies requested them to. “It was no longer necessary to keep workers at times of low production. When there were no orders, the workers stayed at home. When production increased, the women were called back in” (Lima, 2002, p. 112, our translation). The strategy for training and keeping men in sewing was based on the principle of male resistance to the long working hours that the role demands and not running the risk of being away from work due to children, as this role is still a female responsibility. To select men for sewing, companies used the trick of hiring them as sewing technicians. For Lima (2002) it is an unnecessary strategy, because in the absence of jobs and in the face of need, gender resistance loses relevance.

In the sewing workshops in the Seridó region, resistance occurred, but was soon overcome. One interviewee reports that men’s resistance to working in sewing soon dissipated, due to the lack of other opportunities. However, even when men and women are allocated to sewing, discourses and practices are not free from sexist representations. The positions and places occupied by men and women follow socially naturalized determinations as feminine or masculine.

In rural communities, men’s resistance was greater. According to one of the interviewees, some men began to be “seen badly.” The term she used refers to the questions surrounding the sexuality of the young men who entered the sewing industry. But she claims that, with the generalization of jobs in workshops, the male presence was naturalized. The women taught them to sew and no one talks about it anymore. “Today, men have the same ease as women.” In this case, there is a perceived flexibility in social gender roles; however, in rural areas, the demarcation of gender roles is still very rigid.

Sewing machines have woven the development of small towns in the semi-arid Northeast, ensuring full employment. However, this process was accompanied by reports of irregularities and evidence of violations of labor rights and precarious working conditions in the sewing *facções* at the beginning of the activities linked to Pró Sertão.

The president of the seamstresses’ union (Sindiconfecções) of Rio Grande do Norte, comparing Seridó with the capital, Natal, states that salaries are different. In the capital, in addition to the base salary, workers receive productivity bonuses and a health plan, the result of agreements between the union and companies, a practice that does not extend to the interior of the state due to the lack of an agreement with the *facções*. She reports that the number of employees in Natal has been reduced due to the relocation of production by some companies (Reporter Brasil, 2015).

The inspections and complaints filed by Sindconfecções and the Public Ministry of Labor of Rio Grande do Norte were widely reported in the media. The Public Ministry of Labor of Rio Grande do Norte filed a Public Civil Action (ACP 0000694–45.2017.5.21.0007) against the main contracting company. This action was initiated by the Labor Prosecutors’ Group associated with the National Coordination for Combating Fraud (CONAFRET), who conducted inspections of the *facções* and analysed the working conditions and content of the *facções* contracts employed by the company. The action clarifies that it does not question the legality of outsourcing, but rather irregularities involving structural subordination. Around 50 *facções* were inspected by the MPT-RN in 12 municipalities, with workers and *facções* members being interviewed. Several irregularities were found, including the absence of safety measures, inadequate warehouse facilities, excessive working hours and unstable contracts.

The predatory use of outsourcing in companies and workshops, as well as the subordination to a single group that, in some cases, is responsible for 90% of all orders for the *facções*, would make outsourcing fraudulent, since the *facções* would have no autonomy in relation to the group that would effectively manage production.

The subcontracting *facções* used by the main contracting company are adhesion contracts because the micro-subcontractors do not negotiate their terms, which are unilaterally stipulated by the contractor. This includes the price to be paid and the fact that the contract does not provide for prior notice in the event of suspension of the delivery of sewing items.

MPT also denounced the concentration of demand in some companies that, through political relations with their owners, would be favoring several *facções* to the detriment of others, which would have their orders limited seasonally, harming their owners and workers, who would be left without activities and without receiving payment during times of lower demand.

Another problem that should be mentioned is that workshops work towards targets set by companies, and they are not always able to meet them. On the other hand, some companies (*facções*) made large investments expecting a return through increased orders, which did not always happen. This caused some to take out loans to pay labor obligations and others to close with the unilateral termination of commercial relations (MPT-RN).

The testimony of a *facção* owner is categorical regarding cases of rights violations and labor exploitation. He reports that, in the initial years of the workshops’ installation, it was very common for employees’ salaries not to be guaranteed, even if they were formally registered. There was an informal profit-sharing agreement: half went to the employer and the other half was divided among the workers, a division that did not always cover the minimum wage. The interviewee states that this practice has been reduced due to inspections, but it still happens. Another serious issue in the owner’s eyes was the exclusive relationship between the *facção* and the contracting company. Although it is not stated in the formal contract, there are tacit agreements and pressure for the *facção* to work only for a certain company. In times of shortage of orders or recess, workers are laid off or only paid for the days they worked. Inspections now require employees to be paid in full, even if they have not worked due to a lack of orders from contractors.

Due to the increase in the number of *facções*, the quantity of orders received has decreased, always favoring the contractor, which has better negotiation or discarding conditions when convenient^8^. In the interviews and complaints published in the press, the lack of autonomy on the part of the *facções* and the difficulty in negotiating with the contracting companies were evident, in addition to the excessive dependence on a single company. Anxiety and discomfort with inspections, unannounced visits, and demands on production rules and meeting targets make up the daily lives of these *facções*.

In 2017, there were demonstrations by *facção* owners and workers against the Public Ministry of Labor for fear of losing their partnership with companies and, consequently, having to close their business. On the part of the workers, there was fear of losing their jobs. Paradoxical aspects in light of the MPT’s allegations, but understandable in light of a situation without employment and income options.

After 11 years of its creation, Pró-Sertão has shown considerable expansion. Of the 124 workshops that were part of the program in 2024, 40% emerged in the last 5 years, exactly during the pandemic period, and have 12 of their own brands. This period brought great challenges due to the termination of contracts with two large companies that migrated to other areas, generating temporary unemployment, but later recovered with the incentive for the workshops to create their own brands, in addition to the presence of FIERN and SENAI, which began to work in the search for new clients for the sewing workshops. In 2023, the company that is the largest supplier to sewing workshops centralized production in Rio Grande do Norte, based on political agreements. This action directly impacted the workshops, which began to have a greater demand for work from this company. Another thing that has impacted the sector are the agreements and partnerships signed between the State of Rio Grande do Norte and a large Chinese e-commerce fashion retailer that would start producing in Brazil and would have its pieces produced by sewing workshops. Although the agreement was signed in July 2023, the partnerships have not yet materialized (SEDEC-RN, 2024).

### 4.2 Analyses and final considerations

We presented two situations in which a combination of factors may explain a certain positivity in processes of work flexibilization, whether in terms of productive centers marked by self-entrepreneurship and autonomous work, on the one hand; or by outsourcing networks marked by the use of regular salaried work, on the other. Both situations maintain a framework of full employment in their main cities, in a region like the Brazilian northeast, historically marked by droughts that compromise its agricultural activities and by low industrialization, as well as by poverty and misery, only alleviated in recent decades by social policies.

However, a closer look leads us to discuss this positivity or, at least, its limits. The ideology of entrepreneurship has redefined informality in production as flexible work. What was seen as archaic and backward has become synonymous with modernity. Given the dynamics developed in the Hub of Pernambuco Agreste, salaried work, understood as a regular contract, with associated social rights, has for some time now become secondary in the perception of worker-producers. Flexibility sometimes involves being self-employed, sometimes formally employed, but the former is more desirable, with greater possibilities, in a space where formal employment is restricted, making it more interesting to invest in one’s own informal business and face the risks.

It is possible to wonder: to what extent will entrepreneurs in the region resist the new transformations and organizational and technological changes imposed by the dynamics of the global market itself, which favors the territory in terms of competitiveness? Its predominance occurred due to the informal and even illicit nature of the development of its activities. Will entrepreneurs be able to resist the formalization of production and marketing, keeping costs low and, therefore, their competitiveness?

In the case of Rio Grande do Norte, outsourcing networks are mobile, moving around the region in search of lower costs represented by tax incentives and infrastructure. The new production hub, marked by the concentration of *facções* in the Seridó region, serves the interests, mainly, of a large business group that already has factories in Paraguay, as costs are also low in that country, threatening to leave the northeastern state. These *facções* serve other companies that also outsource their production to other countries. How long this regional space in Rio Grande do Norte will remain attractive is unknown. This uncertainty becomes even greater when we consider a recent labor reform that completely freed up outsourcing. The liberation of part-time and temporary contracts, by the 2017 reform, adapts to the production seasonality, with losses for workers who will only earn from the orders in the *facções*. Unlike the Pernambuco hub, regular wages still predominate, perceived as labor relations that provide dignity to the worker. Even working towards goals, the entrepreneurial mindset still does not predominate, but rather the fear of returning to a precarious situation represented by the lack of employment and the impossibility of staying in the region.

The spatial configuration, the territorialization of production resulting from the processes of expansion of capitalism in peripheral regions, “industrializing” areas previously marked by agricultural and livestock production, which went through moments of expansion, such as cotton culture, creates specific dynamics for us to analyze forms of labor subordination. According to Wallerstein (2013), the periphery of the world system provides raw materials and cheap labor. Due to its subordinate position in global markets, productive specialization in primary sectors (commodities), industrial and technological fragility, structural heterogeneity, and the transfer of value, it suffers losses in value in global flows and is overexploited. This situation perpetuates regional, national and international subordination. In Brazil, for example, the north-east can be considered the periphery of the periphery, given that the country’s economic development is primarily concentrated in the south and south-east. In the north-east, the periphery is found in the Agreste and semi-arid regions, which have limited economic activity. Consequently, labor costs are a prominent factor in labor-intensive activities.

In regular workshops, the contract is formal, but workers are tied to seasonal orders from companies, which makes continued employment always uncertain. A common feature of these two situations is the instability of both the market that maintains the autonomous workshops and the large companies that guarantee orders for formal and informal workshops. Furthermore, for these workers, losing their job or business almost necessarily means migrating, given the lack of other options in the studied region.

Both situations are typical of flexible capitalism or the logic of network organization (Castells, 1999), which is characterized by the incorporation of peripheral places into the flows of the globalized economy, in which production incorporates new territories and resignifies them within the logic of cost reduction. “In the course of the history of the globalization of capitalism, much of what is encountered along the way is altered, strained, modified, annulled, mutilated, recreated, or transfigured” (Ianni, 1996, our translation).

Recent productive experiences, in the process of reconstruction of the studied territories, allow us to think of them as linked to the ongoing processes of globalization of the economy, marked by precariousness, flexibility and cost reduction, and in the various paths of globalization. As a multifaceted phenomenon, globalization involves several complexly interrelated dynamics (Santos, 2008). Or as said by Knowles (2014, p. 290), our translation, they are “main and secondary entrances/routes that intersect at all times.”

Formal salaried work, seen from the second half of the 20th century onwards in its positivity in terms of control and limits in the capital-labor relationship, adding rights, establishing working hours, and bringing greater dignity to the worker, has never benefited more than 50% of the workforce in Brazil. In times of crisis, such as the 1990s, formal wage employment reached only 30% of the economically active population, a situation that is also common in much of Latin America. In the Brazilian Northeast, with the exception of the large capitals and public employment, this number was even lower. In any case, regular, registered work, with social rights, has become consolidated in the Brazilian worker’s mindset as a possibility of stability, and access to healthcare and retirement.

However, as we have sought to demonstrate, this ideology remains or develops according to a set of situations that are mobile and that depend on territorial dynamics in which its preponderance is greater or lesser. Thus, in the same region it is possible to find self-employed work, representing full employment regardless of its degree of precariousness, and salaried work, in outsourcing networks with full employment in small municipalities, in which access to rights accompanies their constituent instability.

Entrepreneurs and low-cost wage earners are exemplary cases, through which we can question what brings them together and what drives them apart. On the one hand, autonomous workshops with family and home-based work; on the other, workshops of business groups that also seek to employ family groups, in small towns and rural areas. On the one hand, the positivity of full employment; on the other, precarious and limitless working conditions among entrepreneurs, but with greater income possibilities for workers. In regular workshops, the contract is formal, but deeply marked by the lack of perspective of continuity, given the subordination to orders from the companies. In other words, instability is the rule of employment in workshops, and the market is what keeps them going, guaranteeing orders.

The Northeast began to attract companies by offering tax exemptions, state incentives and low labor costs, particularly in the textile and footwear industries. These situations reflect the precarious nature of modernity brought about by flexible capitalism, which incorporates peripheral regions into the flows of the globalized economy in the name of cost reduction. This also involves the redefinition of places, as defined by Santos (2000). For workers, the choice is to adhere to entrepreneurial logic and accept unstable wages, or migrate in search of new employment and income opportunities to ensure their social reproduction. This involves moving across various territories of precariousness that constitute peripheral capitalism.

The region is characterized by structural precariousness of work marked by low wages, absence of rights, high staff turnover and informality. This precariousness is structural because it is not transitory or caused only by crises; rather, it is inherent in the current form of flexible and peripheral capitalism, constituting a systemic requirement for the valorisation of capital. Indicators of this include the expansion of informal, self-employed or ‘own-account’ work, accompanied by an entrepreneurship ideology; the reduction of labor and social security rights (through reforms that weaken legislation); large-scale subcontracting and outsourcing; the financialisation of life and work, resulting in worker indebtedness; and the platformisation of non-permanent employment. The state plays an important role in this process through its policies to encourage and develop employment with or without ties. This reflects its role as organizer and mediator of capital and labor relations, with capital taking precedence given the country’s class structure.

## Data Availability

The data presented in this article are part of the collection of the Laboratory of Studies on Work, Professions, and Mobilities at the Department of Sociology of UFSCar. Access requests should be sent directly to jacobl@ufscar.br.

## Footnotes

^1^SEBRAE – Serviço de Apoio a Micro e Pequenas Empresas (Brazilian Macro and Small Business Support Service), originally governmental and later private (1990), is a non-profit entity with business and state representation.

^2^According to Milanês (2015, p. 42), in Santa Cruz, since 1975, wooden stalls began to appear to display goods, which were exhibited on the sidewalks and on the ground. It was during the 70s and 80s that Toritama and Caruaru also began to enter the sulanca cycle. “Caruaru, known for its popular fair and handicraft, was the last city, among the three main ones in the Hub, to adopt this line of business” (our translation).

^3^Domestic production units, generally set up inside one of the rooms of the house, such as the living room, bedroom, or garage. They generally work outsourced to *fabricos* and larger factories. But there are also *facções* that have their own brand and sell their goods autonomously.

^4^Local name for small production units, generally made up of members of the same family.

^5^A space outside Moda Center where people set stands on their own, without infrastructure but free from paying the commercial center fees.

^6^Some indicators provide a broader picture of regional precariousness. The average monthly income is R$3,305 in the south-east, R$3,116 in the south, R$3,310 in the centre-west, R$2,211 in the north and R$1,847 in the north-east. The unemployment rate in the north-east is 9%, compared to a national average of 1.7%. The proportion of the population living in extreme poverty is 2.7% in the northeast and 1.7% in Brazil (source: 2024 Report of the Brazilian Observatory of Inequalities).

^7^Emergency aid was a benefit that was established in Brazil by Law No. 13,982/2020, which provided for the transfer of R$0.600 reais per month (initially for 3 months) to informal workers, unemployed, and low-income people, with the aim of reducing the economic impacts caused by the Coronavirus pandemic. As of September 2020, the transfer amount dropped to R$0.300,00.

^8^Due to the large number of workshops, the contracting company can easily discard a contracted workshop as it will always have another to cover the orders.
